# Gut microbiota and rheumatoid arthritis: From pathogenesis to novel therapeutic opportunities

**DOI:** 10.3389/fimmu.2022.1007165

**Published:** 2022-09-08

**Authors:** Ting Zhao, Yuanyuan Wei, Youyang Zhu, Zhaohu Xie, Qingshan Hai, Zhaofu Li, Dongdong Qin

**Affiliations:** ^1^ School of Basic Medical Sciences, Yunnan University of Chinese Medicine, Kunming, China; ^2^ The First School of Clinical Medicine, Yunnan University of Chinese Medicine, Kunming, China; ^3^ The Third Affiliated Hospital, Yunnan University of Chinese Medicine, Kunming, China

**Keywords:** rheumatoid arthritis, gut microbiota, immune response, inflammation, drug treatment

## Abstract

Rheumatoid arthritis (RA) is a chronic autoimmune disease that primarily affects the joints. Microbial infection is considered a crucial inducer of RA. Alterations in the composition of intestinal bacteria in individuals with preclinical and established RA suggest a vital role of the gut microbiota in immune dysfunction characteristic of RA. However, the mechanisms by which gut dysbiosis contributes to RA are not fully understood. Furthermore, multiple therapies commonly used to treat RA may alter gut microbiota diversity, suggesting that modulating the gut microbiota may help prevent or treat RA. Hence, a better understanding of the changes in the gut microbiota that accompany RA should aid the development of novel therapeutic approaches. This mini-review discusses the impact of gut dysbiosis in the pathogenesis of RA, the selection of gut microbiota-related biomarkers for diagnosing RA, and provides examples of cross-modulation between the gut microbiota and some drugs commonly used to treat RA. Some suggestions and outlooks are also raised, which may help guide future research efforts.

## Introduction

Rheumatoid arthritis (RA) is a chronic, immune-mediated disease in which multiple immune cell types and signaling networks misfunction to elicit a maladaptive tissue repair process; this leads to organ damage, predominantly in the vascular system, lungs, and joints (1). RA affects ~1% of the population worldwide (2), and its pathogenesis may be linked to genetic and environmental factors (3, 4). At present, the specific pathogenesis of RA is not well understood.

Humans are one of the most complex microbial ecosystems on the planet, hosting over 100 trillion bacteria, mainly in the distal gut (5). Some gut bacteria species can induce autoimmunity in genetically predisposed animal models (6, 7). Dysbiosis of specific bacterial lineages and alterations in gut microbiota metabolism led to changes in the host immune profile that contribute to RA (8). It has been proposed that the mechanism by which gut microbiota imbalance leads to RA may be related to regulation of immune function by metabolites produced by gut microbes (9, 10). Immune T and B cells have position-specific phenotypes and functions in the mucosa, influenced by the microbiota (11). In turn, bacterial peptidoglycan components are found in the synovial tissue of RA patients, which may contribute to inflammation within the microenvironment of the joint (12, 13). Substantial data published in the past few years demonstrate that an altered composition of the gut microbiota in RA patients is one of the major factors triggering aberrant systemic immunity (14–16). Notably, different strains of gut bacteria can have profoundly different regulatory effects on immune system function. Some strains can stimulate an immune response, benefiting immunocompromised patients, while others can suppress the immune response, affecting immune regulation in RA patients (17–21). For example, segmented filamentous bacteria (SFB) have a unique ability to drive T helper 17 (Th17) cell accumulation in the small intestine’s lamina propria through SFB-derived antigens presented by dendritic cells (22–24). In contrast, the colonization of *Bacteroides fragilis* is associated with enhanced activity of regulatory T cells (Tregs), which may alleviate autoimmune disease (25, 26). Therefore, the relative abundance of different bacterial lineages may lead to changes in the host immune profile and drive inflammatory responses contributing to RA.

This mini-review discusses the role of the gut microbiota in the pathogenesis of RA, summarizes the diagnostic value of gut microbe-based biomarkers, and outlines mutual influences between the gut microbiota and some drugs used to treat RA. Some suggestions and outlooks are also raised to guide future research efforts.

## Role of the gut microbiota in the pathogenesis of RA

Numerous studies highlighted a critical role of the gut microbiota in RA pathogenesis, through mechanisms including mainly production of proinflammatory metabolites, impairment of the intestinal mucosal barrier, and molecular mimicry of autoantigens.

### Inflammatory factors and regulation of the immune response

**Figure 1:**
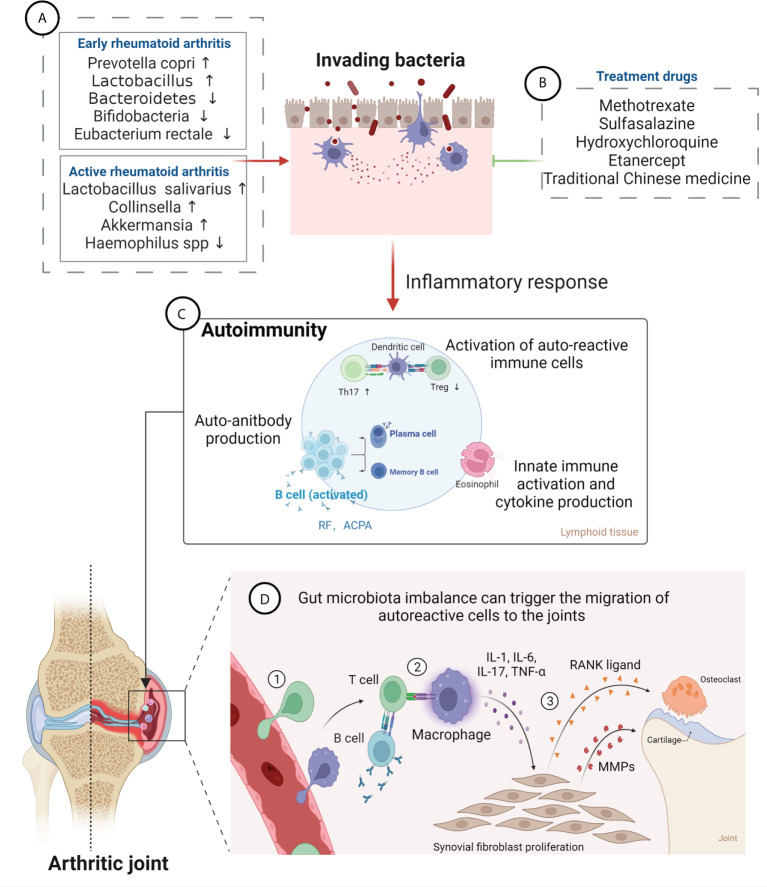
Gut microbiota in the pathogenesis of rheumatoid arthritis (RA) and effects of RA therapeutic drugs on the gut microbiota. **(A)** Changes in the composition of gut microbiota at different stages of RA. Levels of *Prevotella copri* and *Lactobacillus* are increased, while those of *Bacteroidetes, Bifidobacteria and Eubacterium rectale* are decreased, at an early stage; Abundance of *Lactobacillus salivarius*, *Collinsella*, and *Akkermansia* is increased, while that of *Haemophilus* spp. is decreased, in the active RA phase. **(B)** RA treatment drugs can improve gut microbiota imbalance and relieve disease symptoms, mainly including methotrexate, sulfasalazine, hydroxychloroquine, etanercept, and traditional Chinese medicine. **(C)** Gut microbiota can lead to damage of the epithelium and to the opening of the paracellular pathway and can cross the epithelium and get in contact with the immune cells beneath the epithelial layer, which leads to inflammation. Furthermore, bacterial antigens promote activation of autoreactive immune cells (B cell and T cell) in the lymphoid tissues, resulting in an imbalance between regulatory T cells (Tregs) and T helper 17 (Th17) cells, leading to expansion of inflammatory response. Activated B cells produce autoantibodies (anti-citrullinated protein antibody and rheumatoid factor). **(D)** Gut microbiota imbalance can trigger the migration of autoreactive cells to the joints, causing cartilage and bone damage. ① Bacterial antigens trigger promotes inflammation in synovial membrane, attracting leukocytes into the tissue. ② Autoreactive cells activate macrophages, resulting in inflammatory cytokine production. ③ Cytokines induce fibroblasts to produce MMPs (matrix metalloproteinases) and RANKL (receptor activator of nuclear factor κB ligand), which mediate destruction of bone and cartilage tissue, leading to the development of RA.

The gastrointestinal tract hosts the majority of immune cells in the body, with constant interaction with the gut microbiota shaping their function and phenotypes. The gut microbiota mediates constant bidirectional communication with the host immune system in a delicate balance of inducing pathogenic infection or residing in the human body in a commensal state (27). The innate immune cells in the gut-associated lymphoid tissue comprise the first-line of defense against xenobiotics from the gastrointestinal tract. Disturbed gut microbiota can trigger the aberrant activation of innate immune cells, which leads to the upregulation of proinflammatory cytokine including interleukin-12 (IL-12), IL-23, and type I interferons, etc., as well as reduction of anti-inflammatory cytokines including transforming growth factor β and IL-10, etc. (28). Moreover, the adaptive lymphocytes are critical players in autoimmunity, and aberrant activation of T and B cells instigates RA. Gut pathogens with proinflammatory capacities can reshape the immune milieu through innate immune overactivation, followed by aberrant activation of the adaptive immune system. Microbial antigens can be presented to CD4^+^ T cells by dendritic cells and macrophages, leading to differentiation of inflammatory T cell subtypes. Th17 cells are a subset of proinflammatory CD4^+^ T cells characterized by production of interleukin-17 (IL-17) (23). Tregs are also derived from CD4^+^ T cells, show instead immunosuppressive actions, and may inhibit Th17 responses (29, 30). Studies have demonstrated that an increased Th17/Treg ratio is closely related to RA, and that the Th17/Treg balance is strongly regulated by gut microbiota and their metabolites (31, 32). Microbial antigens can also induce overactivation of B lymphocytes with the help of T follicular helper cells, differentiating into plasma cells and producing pathogenic autoantibodies. This may influence the pathogenesis of RA (28). Therefore, the dysbiosis gut microbiota, inflammatory factors, and immune responses are interrelated and jointly affect the development of RA (33) (**Figure 1**).

### Intestinal barrier dysfunction

The intestinal mucosal barrier, formed and maintained by the intestinal epithelium, serves to isolate harmful substances in the intestinal lumen and prevent the invasion of pathogenic antigens. The gut integrity is impaired in RA patients, which leads to the translocation of microbes across the gut barrier into gut tissue and even circulation. Gut microbiota can promote the overactivation of innate and adaptive immunity in the local tissue, resulting in systemic immune dysregulation (28). In addition, disturbed gut microbiota can also trigger the migration of autoreactive cells to the joints, causing local joint inflammation (34). Autoreactive cells activate macrophages, resulting in inflammatory cytokine production. Further, cytokines such as tumor necrosis factor-alpha (TNF-α), IL-6, and IL-1 can induce fibroblasts to produce matrix metalloproteinases and receptor activator of nuclear factor κB ligand, which mediate destruction of the bone and cartilage tissue, leading to the development of RA (35) (**Figure 1**). A study has shown that *Collinsella aerofaciens*, a commensal gut bacterium found to be overrepresented in RA patients, reduces the expression of tight junction proteins in human intestinal epithelial cells and increases disease incidence in HLA-DQ8 transgenic mice subjected to collagen-induced arthritis (CIA) by disrupting the integrity of the intestinal mucosa (36). In contrast, *Faecalibacterium prausnitzii*, a prominent member of the human commensal gut microbiota whose abundance is reduced in patients with RA, was shown to sustain intestinal barrier function, maintain Th17/Treg balance, and exert significant anti-inflammatory effects (37). These findings indicate that changes in gut microbiota diversity may impair intestinal mucosal permeability, facilitating the onset of RA (38, 39). can trigger the migration of autoreactive cells to the joints, causing cartilage and bone damage.

### Molecular mimicry

Molecular mimicry is a mechanism by which pathogen-derived antigens that share sequence homology with self-peptides may lead to cross-activation of autoreactive T or B cells, triggering autoimmunity. Many identical peptides between human tissues and gut microbes bind to HLA-II alleles. The autoimmune candidates have been shown to be enriched in bacterial species belonging to the *Firmicutes* and *Proteobacteria*, which may have a higher disease impact in genetically susceptible individuals (40). N-acetylglucosamine-6-sulfatase and filamin A were identified as T- and B-cell-targeted autoantigens in more than 50% of RA patients (41). The HLA-DR-presented N-acetylglucosamine-6-sulfatase peptide has marked sequence homology with epitopes from sulfatase proteins of the *Prevotella* sp. and *Parabacteroides* sp., whereas the HLA-DR-presented filamin A peptide has homology with epitopes from proteins of the *Prevotella* sp. and *Butyricimonas* sp., another gut commensal (41). In turn, the presence of shared sequences between *Collinsella* and DRB1*0401 suggested that *Collinsella* may induce RA through molecular mimicry (36). These findings thus identify molecular mimicry as a plausible link between disrupted mucosal immune tolerance and systemic immunity in RA patients.

### Other factors

Gut microbiota composition can alter sex hormone levels to affect the occurrence of RA. Sex hormone deficiency increases intestinal permeability, thereby increasing the number of Th17 cells in peripheral blood and the levels of osteoclastogenic cytokines RANKL, IL-17 and TNF-α, promoting bone resorption (42). *Clostridium* can produce enzymes that catalyze the conversion of glucocorticoids into androgens, exerting immunomodulatory effects (43). Implying a strong connection between periodontitis and RA, *Porphyromonas gingivalis*, a major pathogen in periodontitis, promotes citrullination of proteins and production of anti-citrullinated protein antibodies, a hallmark of RA. This is mediated by production of peptidyl arginine deiminase (PAD) and leukotoxin A, which triggers in turn endogenous PAD activation in neutrophils (44). Besides, several metabolites including short-chain fatty acids, serotonin, as well as plant enzymes in the gut can also influence RA (45). However, despite major advances, the mechanisms linking dysbiosis of the gut microbiota and RA remain still incompletely characterized.

## Gut microbiota biomarkers for diagnosing RA

Early diagnosis of RA is critical to provide prompt treatment to slow down joint damage. Scher et al. (2013) reported that the abundance of *Prevotella copri* was increased, while that of *Bacteroidetes* was decreased, in patients with early RA; the presence of *P. copri* was correlated with a reduction in the abundance of other bacterial groups, including many beneficial microbes (46). Transplanting gut microbiota (with a high abundance of *Prevotella*) from patients with early RA into germ-free SKG mice can induce severe arthritis (47). In addition, increased number and species of intestinal *Lactobacillus* were observed in early RA patients than in healthy individuals (48). Studies have shown that the monocontamination of germ-free IL-1 receptor antagonist–deficient (IL1rn^-^/^-^) mice, which develop spontaneous arthritis due to excessive IL-1 signaling, with indigenous *Lactobacillus bifidus* resulted in rapid onset of arthritis that reached incidence rate and severity scores comparable to those recorded in non–germ-free mice (49). It was in turn reported that in early RA patients the fecal microbiota contained significantly less *Bifidobacteria*, *B. fragilis*, and *Eubacterium rectale* than in patients with fibromyalgia (50).

In the active period of RA, depletion of *Haemophilus* spp. in the patients’ gut, teeth, and saliva correlated negatively with RA severity and serum antibody levels. In contrast, *Lactobacillus salivarius* was present in increased amounts in cases of very active RA (16). Meanwhile, gut microbiota analysis revealed higher relative abundances of the genera *Collinsella* and *Akkermansia* in patients with active, compared to inactive, RA disease status (51). A study indicated that treatment with *Collinsella* exacerbated CIA in HLA-DQ8 transgenic mice (36). These observations reaffirm the impact of alterations in the gut microbiota on RA severity and suggest that changes in gut microbiota composition may serve as markers for the diagnosis of RA. However, further research is needed to conclusively identify reliable gut microbiota biomarkers for diagnosing RA (**Figure 1**).

## Therapeutic modulation of the gut microbiota and impact of gut microbes on the efficacy of RA drugs

The gut microbiota was found to predict drug response in RA (52). On the other hand, in recent years oral probiotics and fecal flora transplantation have shown promising results when used as adjuvant therapy for treating RA, by directly and indirectly modulating the gut microbiota. Indirect regulation of gut microbiota in RA patients and animal models is also exerted by disease-modifying anti-rheumatic drugs (DMARDs), traditional Chinese medicine (TCM) herbs and prescriptions, and by adjusting diet structure.

### Direct regulation of gut microbiota

Probiotics, defined as “live microorganisms that, when administered in adequate amounts, confer a health benefit on the host” (53), can reduce the abundance of pathogenic bacteria by competing for nutrition and colonization sites. At the same time, probiotics can alleviate RA symptoms by producing antibiotics and strengthening the intestinal barrier, with beneficial modulation of the immune function (54). Studies conducted in rats with adjuvant-induced arthritis (AIA) showed that oral administration of *L. casei* or *L. acidophilus* reduced arthritic inflammation, pannus formation, and cartilage destruction (55–57). More recently, it was reported that administration of *L. casei* to AIA rats significantly suppressed arthritis and protected against bone loss by reducing dysbiosis of the gut microbiota (58). However, supplementation with *L. reuteri* and *L. rhamnosus* GG did not significantly reduce RA disease activity (59, 60), suggesting that different *Lactobacillus* species may act differently on RA.

Fecal microbiota transplantation (FMT) refers to the introduction of gut microbiota obtained from the feces of a healthy donor into a patient’s gastrointestinal tract (61). Normalizing the gut microbiota through FMT may potentially improve RA symptoms. A case of a patient with refractory RA successfully treated with FMT indicated that FMT may have an excellent therapeutic effect on RA (62). However, clinical studies examining the efficacy of FMT in RA patients are so far scarce.

### Indirect regulation of gut microbiota

DMARDs may indirectly affect and remodel the structure and function of gut microbiota to regulate systemic immunity. Studies have demonstrated that microbial differences in the gastrointestinal tract of RA patients may partially determine the bioavailability and subsequent clinical outcome of methotrexate (63, 64). In turn, methotrexate treatment was shown to partially restore normal gut microbiota composition in RA patients (16, 65). Thus, the gut microbiota may be a predictor of clinical response to methotrexate, influencing the treatment response rate.

Another DMARD, i.e. sulfasalazine, is cleaved by the action of bacterial azoreductases in the large intestine into sulfapyridine and mesalazine (66, 67). Sulfapyridine affects the immune system and appears to normalize lymphocyte activity by regulating gut microbiota (68). A study in active RA patients showed that sulfasalazine therapy led to a substantial fall in fecal counts of *Clostridium perfringens* and *Escherichia coli* (69). Furthermore, sulfasalazine treatment significantly altered fecal microflora of RA patients by reducing total aerobic bacteria, *Bacteroides*, and *Escherichia coli*, and increasing the numbers of *Bacillus* (70). However, thorough characterization of the effects of sulfasalazine therapy on the gut microbiota in RA is still lacking.

Treatment with etanercept, a TNF-α antagonist, was shown to beneficially impact gut microbiota composition. In RA patients, etanercept treatment was associated with enrichment of *Cyanobacteria*, including members of the *Nostocophycideae* class and the *Nostocales* order (which were not represented among naïve patients), as well as with decreased abundance of *Clostridiaceae* and *Deltaproteobacteria* (71). In CIA mice, etanercept treatment led to decreased microbial community richness and diversity, increasing the abundance of *Escherichia* and *Shigella* and decreasing the abundance of *Clostridium XIVa*, *Tannerella*, and *Lactobacillus* (72). In contrast, hydroxychloroquine treatment in RA patients was associated with increased intestinal bacterial richness and diversity, suggestive of restoration of normal microbiota. In addition, the abundance of *Faecalibacterium*, found to be decreased prior to treatment, was positively correlated with the use of hydroxychloroquine (36, 71).

Numerous studies indicated that TCM-based therapies provide significant curative effects and elicit minor adverse reactions in RA (73). Notably, part of the beneficial effects of TCM on RA may be ascribed to regulation of the intestinal flora. Mei et al. (2021) showed that depletion of *Clostridium celatum* in RA patients could be reversed by treatment with the Huayu-Qiangshen-Tongbi formula (74). Administration of total glucosides of paeony to CIA rats corrected 78% of the taxonomic differences in microbial structure, while also increasing the relative abundance of certain forms of beneficial commensal bacteria (75). Similarly, most of the 19 types of bacteria found to be altered at the family level in CIA rats could be regulated by the Zushima tablet (76). It was also reported that Qingluo Tongbi decoction can effectively ameliorate arthritis in AIA rats at least partly by decreasing inflammatory responses regulated by the gut microbiota (77).

In addition to therapeutic drugs, dietary nutrients also affect the composition and function of the gut microbiota and may thus have an important impact on the prevention and treatment of RA. Dietary fiber, abundant in vegetarian diets, can improve gut microbiota composition in RA patients and reduce joint pain (78). Reducing the intake of carbohydrates can help improve the balance of intestinal flora and immune function (79, 80). The omega-3 polyunsaturated fatty acids can help to maintain the intestinal barrier integrity and interact with host immune cells (81). A low ratio of omega-3/omega-6 fatty acids has been shown to promote inflammation, increasing the risk of RA (82, 83). Increased sodium intake can also increase the risk of RA (84). Studies have confirmed that a high-salt diet may lead to dysbiosis of gut microbiota, which promotes the micro-inflammatory state and autoimmune processes (85, 86). Indeed, several clinical trials have demonstrated that RA severity can be alleviated through dietary interventions (87). Thus, a better understanding of the mutual influences between the gut microbiota and some drugs or dietary nutrients will help to achieve optimal therapeutic effects for RA.

## Perspectives and conclusion

After decades of research, the fundamental role of the gut microbiota in health and disease is now firmly established. It is thus widely recognized that the gut microbiota can affect almost all aspects of the host, and its dysregulation is associated with dysregulated immune tolerance and RA development. Indeed, changes in the gut microbiota can precede the onset of RA and are closely related to disease activity afterwards. Analysis of gut microbiota composition can also predict susceptibility to RA, and has become a useful method to predict and control RA incidence. Furthermore, the human gut microbiota and their enzymatic products can affect the bioavailability, clinical efficacy, and toxicity of a wide array of drugs through direct and indirect mechanisms. Conversely, various medicines and active ingredients modulate immune cell function by normalizing the composition of the gut microbiota. Although significant variations in some specific microbial communities have been detected in association with RA, further research is needed to clarify the role of the gut microbiota in RA and its impact on the mechanisms of action and efficacy of DMARDs. Notwithstanding, mounting evidence indicating that targeted modulation of the gut microbiota may alleviate RA suggests that personalized treatment approaches based on patient microbiome profiles may increase drug efficacy, lower toxicity risk, and improve clinical outcome.

## Author contributions

All authors listed have made a substantial, direct and intellectual contribution to the work, and approved it for publication.

## Funding

National Natural Science Foundation of China (31960178, 82160923, 81960863, 82160901, and 81960870); Construction Project of National TCM Clinical Research Base (2018 No. 131); Yunnan Provincial Fund for Medical Research Center: Clinical Evaluation and Basic Research on the Treatment of rheumatoid arthritis and gout by TCM (202102AA310006); Clinical Trial for the Treatment of Rheumatoid Arthritis with Warming yang and Smoothening Meridians (201507001-07, registration number: ChiCTR-INR-16010290); Clinical Cooperative Project of Chinese and Western Medicine for Major and Knotty Diseases; Yunnan Provincial Key Laboratory Construction Project Funding; Yunnan Provincial Key Laboratory of Chinese Medicine Rheumatology and Immunology; Yunnan Provincial Ten Thousands Program Famous Doctor Special; Yunnan Province Qingguo Wang Expert Workstation Construction Project (202005AF150017); Yunnan Applied Basic Research Projects- Union Foundation [2019FF002(-031)]; Applied Basic Research Programs of Science and Technology Commission Foundation of Yunnan Province (2019FA007); Key Laboratory of Traditional Chinese Medicine for Prevention and Treatment of Neuropsychiatric Diseases, Yunnan Provincial Department of Education; Scientific Research Projects for High-level Talents of Yunnan University of Chinese Medicine (2019YZG01); Young Top-Notch Talent in 10,000 Talent Program of Yunnan Province (YNWR-QNBJ-2019-235).

## Conflict of interest

The authors declare that the research was conducted in the absence of any commercial or financial relationships that could be construed as a potential conflict of interest.

## Publisher’s note

All claims expressed in this article are solely those of the authors and do not necessarily represent those of their affiliated organizations, or those of the publisher, the editors and the reviewers. Any product that may be evaluated in this article, or claim that may be made by its manufacturer, is not guaranteed or endorsed by the publisher.
